# Resting-State Functional Connectivity Impairment in Patients with Major Depressive Episode

**DOI:** 10.3390/ijerph192114045

**Published:** 2022-10-28

**Authors:** Drozdstoy Stoyanov, Vladimir Khorev, Rositsa Paunova, Sevdalina Kandilarova, Denitsa Simeonova, Artem Badarin, Alexander Hramov, Semen Kurkin

**Affiliations:** 1 Department of Psychiatry and Medical Psychology, Research Institute, Medical University Plovdiv, 4002 Plovdiv, Bulgaria; 2 Baltic Center for Artificial Intelligence and Neurotechnology, Immanuel Kant Baltic Federal University, 236041 Kaliningrad, Russia; 3 Neuroscience Research Institute, Samara State Medical University, 443001 Samara, Russia

**Keywords:** functional connectivity, functional magnetic-resonance imaging, resting state, mood disorders, classification

## Abstract

Aim: This study aims to develop new approaches to characterize brain networks to potentially contribute to a better understanding of mechanisms involved in depression. Method and subjects: We recruited 90 subjects: 49 healthy controls (HC) and 41 patients with a major depressive episode (MDE). All subjects underwent clinical evaluation and functional resting-state MRI. The data were processed investigating functional connectivity network measures across the two groups using Brain Connectivity Toolbox. The statistical inferences were developed at a functional network level, using a false discovery rate method. Linear discriminant analysis was used to differentiate between the two groups. Results and discussion: Significant differences in functional connectivity (FC) between depressed patients vs. healthy controls was demonstrated, with brain regions including the lingual gyrus, cerebellum, midcingulate cortex and thalamus more prominent in healthy subjects as compared to depression where the orbitofrontal cortex emerged as a key node. Linear discriminant analysis demonstrated that full-connectivity matrices were the most precise in differentiating between depression vs. health subjects. Conclusion: The study provides supportive evidence for impaired functional connectivity networks in MDE patients.

## 1. Introduction

Major depressive disorder (MDD) is a leading cause of disability worldwide [1]. However, its exact pathophysiological mechanism remains unclear. Symptoms of depressive episode include sadness, anhedonia, insomnia, restlessness, and suicidal thoughts [2]. Moreover, this condition is characterized by impairments in cognitive and emotional processing [3]. There is evidence suggesting that cognitive dysfunction could be seen not only in the acute phase but in remission as well, and it could even worsen the condition’s outcome [4,5].

Brain networks can be defined as a set of regions that exhibit correlated activity in resting-state condition or during task performance [6,7]. In recent studies, researchers concentrated on building brain functional networks, as well as searching for abnormal communication between them in order to elucidate the pathophysiological mechanisms of MDD [8]. Different methods are used for constructing brain networks such as region of interest (ROI) analysis, seed-based analysis or independent component analysis (ICA) with the first two being preferred in hypothesis driven research; while the latter is an example of data driven approach [9]. However, for each of these methods there are strengths and weaknesses. In the ROI-based method the definition of the regions could affect functional connectivity patterns [10]. Moreover, seed-based analysis results depend much on the positioning of the seed voxel, which could lead to inconsistent results [11]. As a data-driven approach, ICA extracts the signal and creates a number of components (limited by the researcher), which could affect the number of spatially distinct networks and is dependent on the knowledge and critical thinking of the investigator for the interpretation.

Generally, the brain networks which are thought to be involved in depression are the default mode network (DMN), salience network (SN), and executive control network (ECN) [12,13,14]. The rostral medial prefrontal cortex (rmPFC) is a key node in the DMN and has been reported to support socio-cognitive and socio-affective processes which are impaired in patients with major depressive disorder [15]. Moreover, decreased thalamic connectivity within the SN has been reported in patients with major depressive disorder [12]. However, other brain regions are affected by the major depressive disorder as well. Patients with MDD had increased connectivity between the right anterior hippocampus (rAHipp) and lingual gyrus (LinG) [16]. In addition, there was also a decreased connectivity between the right posterior hippocampus (rPHipp) and right inferior frontal gyrus (rIFG) [16]. On the other hand, decreased connections between the frontoparietal network and subcortical network and increased connections between the frontoparietal network and salience network were reported, which shows the dysregulated neuronal activity in patients with MDD [17].

Understanding brain network dysfunctions in depression is a promising key in the process of elucidating the pathophysiological mechanisms involved. Previous studies have used amplitudes of low-frequency fluctuations and resting-state fMRI data in order to observe the connections between DMN and CEN [18]. A large study by Liang et al. managed to divide two subgroups of MDD according to their hyper and hypo DMN connectivity [19]. They proposed that the hypo-DMN function relates to the age-related severity of depressive symptoms. Bhaskar Sen et al. proposed a different methodology for predicting the chance of suffering from depression by examining connectivity values of different brain regions during resting-state fMRI [20]. In addition, a multivariate approach was implemented to differentiate between MDD and healthy controls, where not only cross-network connections were found but also the supramarginal gyrus appeared to be the most discriminative one [21].

In contrast to the abundance of classical functional connectivity research, there are very few studies which investigate the pathology of psychiatric conditions from the point of graph-theory analysis and its characteristics such as node strength, centrality, etc. According to Jacob et al. MDD patients showed decreased node strength of the right hippocampus and decreased clustering coefficient of the right dentate gyrus in contrast to the HC group [22]. In another study using this method MDD patients exhibited reduced centrality in parietal lobule, lingual gyrus and thalamus and there was node disruption in brain connectivity of the patients which correlated with their depressive symptoms and cognitive performance [23].

Moreover, a meta-analysis from 2017 shows the role of ACC, as part of SN, in differentiating drug-naive and medicated patients with MDD [24]. On the other hand, meta-analysis reveals an aberrant intrinsic brain activity predominantly in the insula, medial prefrontal cortex and cerebellum [25]. In addition, according to a meta-analysis research study it reported that the resting-state fMRI could be used for the classification of MDD and HC with 85% sensitivity, 83% specificity according to the methodology [26].

Our approach is an explorative, data-driven, semi-unsupervised study aiming to investigate newer approaches to characterize brain networks measures in terms of clustering coefficient, node centrality, and node strength. The purpose of this approach is to address network generative principles, order dependencies and the community structure of networks, which could potentially contribute to a better understanding of the mechanisms involved in depression and in certain perspectives differentiate patients with depression from healthy individuals, aiding the diagnostic process.

## 2. Subjects and Methods

### 2.1. Subjects

We recruited 90 subjects: 49 healthy controls (HC) and 41 patients with a major depressive episode (MDE) in the context of major depressive disorder (*n* = 35) or bipolar affective disorder (*n* = 6). Subjects from both groups were assessed by experienced psychiatrists using Mini International Neuropsychiatric Interview [27] and Montgomery–Åsberg Depression Rating Scale (MADRS) [28]. Subjects having a previous history of comorbid (for HC and patients, respectively) psychiatric conditions, autoimmune diseases, neurological diseases, history of head trauma, or any metal implants incompatible with the MRI were excluded. All participants provided a written consent form complying with the Declaration of Helsinki. The study was approved by the Medical University of Plovdiv Ethical Committee (2/19 April 2018).

#### Demographic and Clinical Characteristics

The two groups of subjects did not differ significantly in terms of mean age, sex, and level of education distributions. Expectedly, patients had significantly higher MADRS scores (see Table 1).

### 2.2. Methods

#### 2.2.1. MR Scanning

The MR scanning procedure was performed on a 3T MRI system (GE Discovery 750w, General Electric, Boston, MA, USA). The protocol included a high-resolution structural scan (Sag 3D T1) with slice thickness of 1 mm, matrix 256 × 256, TR (relaxation time) 7.2 s, TE (echo time) 2.3 s, and flip angle 12°, FOV 24, 368 slices and resting-state functional scan—2D echo-planar imaging (EPI), with slice thickness 3 mm, matrix 64 × 64, TR 2000 ms, TE 30 ms, 36 slices, flip angle 90°, FOV 24, a total of 192 volumes. Before the EPI sequence, subjects were instructed to remain as still as possible with their eyes closed and not to think of anything in particular.

#### 2.2.2. Image Processing

Neuroimaging data were processed using SPM 12 software (Statistical Parametric Mapping, http://www.fil.ion.ucl.ac.uk/spm/, accessed on 4 October 2021) running on MATLAB R2021 for Windows. The functional images of each participant were first realigned, co-registered with the high-resolution anatomical image, and normalized to standard MNI space. Parameters for the realignment step were the following: quality 0.9, separation 4, smoothing FWHM 5, 2-nd degree B-spline interpolation, no wrap, 12 × 12 basis function, regularization 1 with medium factor, without Jacobian deformations, 5 iterations, average Taylor expansion point. The co-registration method was set to the normalized mutual information with the following parameters: separation [4 2], tolerances [0.02 0.02 0.02 0.001 0.001 0.001 0.01 0.01 0.01 0.001 0.001 0.001], histogram smoothing [7 7]. MNI normalization parameters were the following: bias regularization 0.0001, bias FWHM 60mm cutoff, affine regularization ICBM European brain template, warping regularization [0 0.001 0.5 0.05 0.2], no smoothing, sampling distance 3.

#### 2.2.3. Connectivity Matrix Calculation

The normalized functional MRI volumes extracted with the help of SPM12 and the “spm_data_read” function were parcellated into 166 regions according to the automated anatomical labeling atlas AAL3 [29]. We have chosen the AAL atlas because it is the most commonly used parcellation scheme in functional network studies [30]. To estimate the connectivity between the regions of interest, we calculated an average BOLD time series *x_i_*(*t*) (across the voxels in each parcellation *i*) and Pearson correlation coefficients for all pairs of the mean parcellation activities. The Pearson correlation coefficient measures the linear relationship between two random variables and is good for low-frequency processes, which also include fMRI [31]. Connectivity Matrix calculation from the averaged activity time series was performed with the help of Matlab statistics “corrcoef” function. Thus, we obtained for each subject a 166 × 166 symmetric connectivity matrix R. Each cell of the connectivity matrix (*r_i,j_*) represents the strength of the connection (or edge) between two parcels:

(1) $$r_{i,j}=\frac{\sum_{k=1}^{n}\left(x_{i,k}-{\bar{x}}_{i})(x_{j,k}-{\bar{x}}_{j}\right)}{\sqrt{\sum_{k=1}^{n}{\left(x_{i,k}-{\bar{x}}_{i}\right)}^{2}\sum_{k=1}^{n}{\left(x_{j,k}-{\bar{x}}_{j}\right)}^{2}}}$$

Here, *n* is the length of the *x* time series, and $\bar{x}$ is the mean of the *x* time series.

#### 2.2.4. Network Measure

To characterize the identified brain networks with meaningful and simplistic computable indicators, we used the following measures implemented in the Brain Connectivity Toolbox [32,33]:

(1) The weighted undirected clustering coefficient [34], which is typically used as a measure of the prevalence of node clusters in a network. For the particular node *i* local undirected clustering coefficient of the network can be calculated as follows:

(2) $$C_{clust,i}=\frac{1}{l_{i}(l_{i}-1)}r_{i,j}r_{j,k}r_{k,i},\;i,j,k\in [1..166]$$

where $l_{i}=\sum_{j=1}^{166}r_{i,j}$ is the total weight of the relationship of the *i*-th node, 166 is the number of regions. The mean value for the whole network will be:

(3) $$<C_{clust}>=\sum_{i}C_{clust,i}$$

(2) The weighted undirected eigenvector centrality [33,35], which measures a node’s importance while considering the importance of its neighbors. This measure is defined as the eigenvector, *v*, associated with the largest eigenvalue *λ* of the correlation matrix, and can be written as:

(4) $$\theta_{i}=\frac{1}{\lambda_{i}}\sum_{j=1}^{166}r_{i,j}v_{j}$$

In analogy with (3), the mean centrality for the network will be denoted as <*ϴ*>.

(3) The node strength [32], which is the measure representing the sum of the edge weights:

(5) $$Ns_{i}=\sum_{j=1}^{166}r_{i,j}$$

In analogy with (3), the mean node strength for the network will be denoted as <*Ns*>.

These measures address network generative principles, order dependencies, and community structure of networks. These measures are implemented as a diagnostic tool for explicitly linking micro-scale features of network organization to macro-scale characteristics of neurophysiological dynamics [33].

### 2.3. Statistical Analysis

Statistical analysis of the demographic and clinical characteristics of the participants were performed using IBM SPSS 28.0 for Windows. The significance level was set to *p* < 0.05. We employed the Chi-square test for the categorical variables (sex and education). We tested the normality of the distributions of the continuous variables with the Kolmogorov–Smirnov one-sample criterion, which did not verify the normality for age and MADRS scores. In that regard, we employed the two-sample Kolmogorov–Smirnov nonparametric test to assess the differences between the control and patient groups.

The false discovery rate (FDR) method [36] with t-test implemented in NBS software [37] was used to assess the differences between the obtained correlation matrices for the two groups (control vs. patients) with a significance level of *p* = 0.0001 and 100,000 permutations. We included diagnoses as dummy-coded covariates in the *t*-test to control the presence of two depressive subgroups (MDD and bipolar).

For independent samples t-test was performed on the network mean values <*C_clust_*>, <*ϴ*>, <*Ns*>. Obtained network measures were tested for being normally distributed with the Kolmogorov–Smirnov one-sample criterion.

We applied permutation-based statistical testing [38] to test the significance difference (control vs. patients) between the distributions of the network measures by nodes. We revealed the node clusters, which were significantly different between healthy controls and depression groups based on their local values of *C_clust,i_*, *ϴ_i_*, *Ns_i_*. Order in the procedure is defined by the neighborhood of the anatomical parcellations, this allows us to analyze the spatial structures of the obtained clusters. For the visualization of the network structures, we used the BrainNet Viewer [39].

#### Linear Discriminant Analysis

To evaluate the diagnostic value of the identified differences in functional networks between the groups, we applied a linear discriminant analysis (LDA) (using the Classification Learner toolbox, Matlab) to data feature vectors from the control and depression groups. Linear discriminant analysis is widely used for diagnostic purposes; the multivariate supervised classification method sorts objects of the study into groups by finding linear combinations of a number of variables [40]. The tested variants of feature vectors included: full functional connectivity (FC) matrices, FC matrices with only connections that were considered significant through the FDR analysis, node clustering coefficients, node strength, and node centrality. Statistical significance of the results was ensured by the use of the nested k-fold (k = 10) cross-validation with each test run 1000 times. Names of classes used in the algorithm for training and classification were specified as a categorical set including “control” and “depression”. Cost of misclassification of a point was set to the default Cost(*i*,*j*) = 1 if *i* ≠ *j*, and Cost(*i*,*j*) = 0 if *i* = *j*. A linear coefficient threshold was set to zero. The discriminant type was set, recommended by the toolbox “pseudolinear” type to avoid problems with zeros and negative values in the predictors set. Other parameters including the enforced amount of regularization or prior probabilities were not applied. The chance level of the LDA for the considered problem is 50%.

## 3. Results

### 3.1. Connectivity Analysis

**Figure 1:**
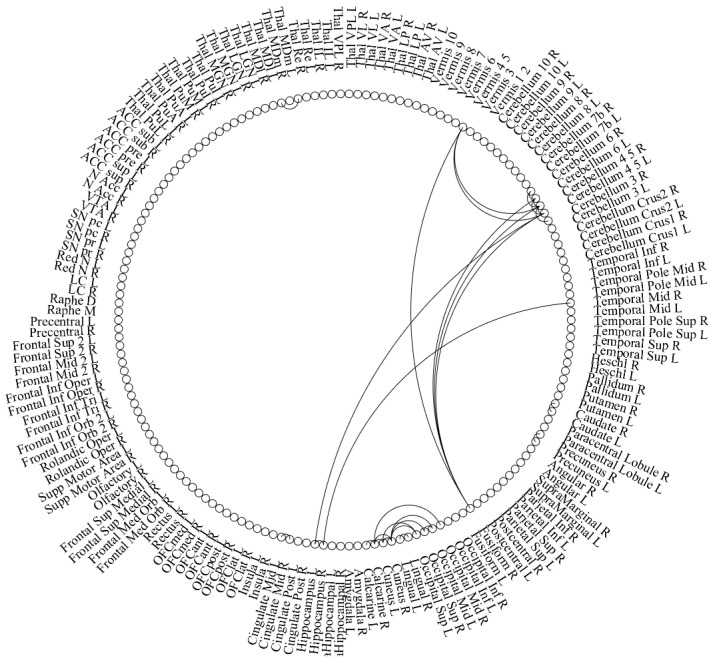
Illustration of the top 30 significant connections were stronger in the control group compared to the depression group.

Our analysis identified a total of 2673 connections and 152 nodes, which were significantly stronger in the control group compared to the depression group. We present the top 30 of those connections in Table 2 and in Figure 1.

### 3.2. Network Analysis

The Kolmogorov–Smirnov one-sample criterion confirmed the normality of the mean network measures processed with the t-test. Mean values for the node strength <*Ns*> (t_2, 40_= 0.179; *p* = 0.8584), node eigenvector centrality <*ϴ*> (t_2, 40_ = 0.004; *p* = 0.9993), and the clustering coefficient <*C_clust_*> (t_2, 40_ = 0.398; *p* = 0.5351) did not show a significant difference between the two groups.

Permutation-based statistical testing revealed 5 positive and 2 negative clusters of nodes in the distribution of node eigenvector centrality; 4 positive and 1 negative clusters in the distribution of node strength and 2 positive and 1 negative clusters in the distribution of clustering coefficient (see Table 3, Table 4 and Table 5). A positive cluster corresponds to a set of neighboring nodes in which the corresponding network measure is significantly higher in the control group, while for a negative cluster, the network measure is significantly higher in the MDE group.

**Figure 2:**
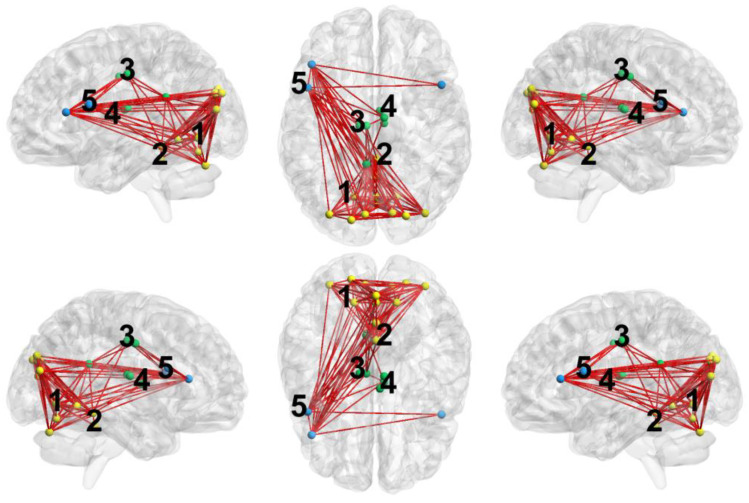
Connected graph obtained for the significant positive clusters (Control > MDE) of the considered network measures. Dots correspond to the nodes of the clusters and lines correspond to the significant connections between the nodes. Numbers denote approximate positions of the clusters (see Table 3). Yellow clusters are similar for all considered network measures; green clusters are similar for eigenvector centrality and node strength; blue clusters are unique for eigenvector centrality.

**Figure 3:**
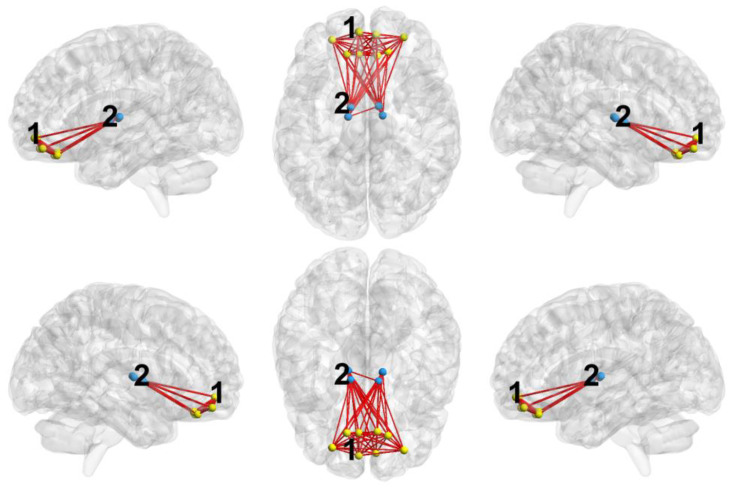
Connected graph obtained for the significant negative clusters (MDE > Control) of the considered network measures. Dots correspond to the nodes of the clusters and lines correspond to the significant connections between the nodes. Numbers denote approximate positions of the clusters (see Table 3). Yellow clusters are similar for all considered network measures and blue clusters are unique for eigenvector centrality.

To construct specific networks, we considered only nodes included in the significant clusters (separately for positive and negative ones) and only significant connections between such nodes that were found in the previous step with the FDR method. Specific networks for the significant positive clusters for all considered network measures are presented in a unified manner in Figure 2, and the specific networks for negative clusters are shown in Figure 3; Table A1 and Table A2 in the Appendix A contain information about all connections belonging to these networks. The first two positive clusters are very similar for all network measures considered, the 2nd and 3rd are for eigenvector centrality and node strength, and the 5th is unique for eigenvector centrality. The first negative cluster is similar for all network measures considered and the 2nd is unique for eigenvector centrality.

For the comparison Control > MDE, the main hubs common for the considered network measures are the lingual gyrus, the superior occipital gyrus, and the middle occipital gyrus.

Moreover, Clusters 2–4 are common for the node centrality and node strength indicators.

### 3.3. Linear Discriminant Analysis (LDA)

**Figure 4:**
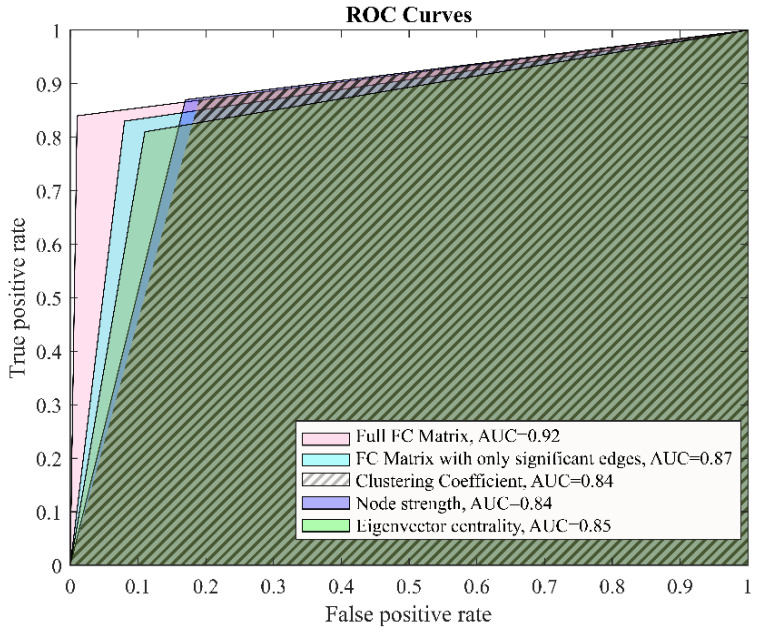
Receiver operating characteristic for the LDA classifiers for different feature vectors, AUC = area under curve.

Employing LDA, we explored the accuracy of the diagnostic classification based on different connectivity measures. As seen in Table 6, all methods were suitable for classification purposes (precision of at least 80%), although the best results obtained from the LDA were full functional connectivity matrices. An illustration of the results are given in Figure 4, where the ROC curves are presented.

## 4. Discussion

Our current study resulted in the following major findings: (1) patients with depression demonstrated decreased functional connectivity within as well as between different brain regions such as precuneus (PreCu), cuneus (Cu), superior occipital gyrus (SOG), lingual gyrus (LG), fusiform gyrus (FG), cerebellum, along with limbic structures including the hippocampus (Hipp) and cingulate gyrus; (2) two positive clusters (with higher measures in HC as compared to MDE patients) were common for all three network measures including node eigenvector centrality, node strength and clustering coefficient (the first cluster included mainly occipital brain regions—Cu, LG, middle and SOG while the second encompassed parts of the vermis); (3) another two positive clusters were common for both centrality and strength measures (the first one—middle cingulum bilaterally and left posterior cingulum, the second one—right anteroventral and bilateral lateroposterior thalamus); (4) one negative cluster (higher in MDE group) encompassing mainly orbitofrontal regions was common for all network characteristics while another one (with thalamic nodes) was featured solely by eigenvector centrality; (5) the LDA demonstrated that the full-connectivity matrices had the highest precision in differentiating between depression and health whilst the clustering coefficient was the least suitable measure. The significance of these findings will be discussed in the following lines.

The most significantly different connection was the one between the left and right PreCu. The function of the precuneus at rest is traditionally linked to the default mode network (DMN), which is responsible for internally oriented attention and self-reference [41]. In this context, the current findings of changed connectivity within the PreCu is not surprising and is seen in many resting-state studies of depression where the DMN, as well as its subregions, is found to be hyperactivated and hyperconnected [42,43]. This is usually linked to the clinical features of depression as a state of increased internalization (including ruminations) [44]. Notably, the direction of the change in our sample is opposite (hypoconnectivity between left and right PreCu) which is in line with the most recent meta-analytic studies finding reduced DMN connectivity especially in recurrent depression as is the case of our patient group [45].

We found no significant difference in the global (network-wide averaged) eigenvector centrality, node strength, and clustering coefficient measures between the control and MDE groups. Thus, in terms of global network topology, the characteristic functional networks do not differ between the groups under consideration. The main differences are observed at the local level, as indicated by the significant clusters in the network measure distributions (see Table 3, Table 4 and Table 5).

The first two positive clusters (Control > MDE) in the network measure distributions, in which eigenvector centrality, node strength, and clustering coefficient are higher in the control group, include bilateral lingual, superior, and middle occipital gyri and cerebellar regions (vermis). In terms of the network measures, a higher local clustering coefficient means that short-range (local) connections prevail over long-range (global) ones in these brain areas in the control group, and network clusters are formed there. A higher eigenvector centrality and node strength in the same nodes in the control group corresponds to a stronger integration of emerging clusters in the network and stronger connections of these nodes (hubs) with other large hubs. The remaining three positive clusters (the cingulate, thalamic nodes and the inferior frontal gyrus) are characterized by increased eigenvector centrality and node strength (only for the 3rd and 4th clusters) in the control group. This indicates that these nodes form larger and more strong integrative network hubs compared with the MDE group. These conclusions are also supported qualitatively in Figure 2.

Of note, all considered measures pointed to the role of the left LG as a major hub in the occipital cluster, with a number of connections demonstrating a significant difference in the depressed as compared to healthy individuals. In accordance with these results, depression has been linked to impaired static and dynamic FC of the LG. The role of the left LG in depression has been reported in a most recent functional connectivity study assessing the effect of childhood trauma [46]. The authors report that the FC changes of the left LG were not affected by the presence or absence of traumatic events, which may reflect a general vulnerability to depression. In support of this notion is a recent study by Wang et al. on electroconvulsive treatment of depression. The authors found changes in functional connectivity of the lingual gyrus to be persistent before as well as after the procedures [47]. The other common hub for all three network measures in our study was the vermis. Apart from the well-known motor functions, there is growing evidence for the involvement of different parts of the vermis in higher order functions including cognitive and emotional processing [48]. Although long neglected, the role of the cerebellum in emotion has been now reestablished also in a recent consensus paper [49].

The links with psychiatric disorders are not surprising. Cerebellar gray matter reductions as well as decreased activity and connectivity of this region have been reported in depression [25,50]. Some authors proposed that impaired cerebellar function contributes to abnormalities in predictive coding and homeostatic dysregulation in depressive disorder [51].

The third cluster which demonstrated significantly different distribution of node strength and node centrality encompassed the mid-cingulate cortex(MCC) which is divided into the posterior MCC involved in multisensory orientation of the head and body in space while the anterior MCC is involved in action-reinforcement associations and selection based on the amount of reward or aversive properties of a potential movement. MCC contributes to cognitive control and decision making [52]. The anterior subregion also has high dopaminergic afferents and high dopamine-1 receptor binding and is engaged in reward processes [53]. Emotional n-back tasks elicited differential activation of the posterior MCC in unipolar compared to bipolar depression [54]. In addition, MDD patients as compared to healthy individuals failed to activate MCC when an emotional stimulus was paired by a neutral one [55].

The fourth positive cluster yielded by the comparison HC vs. MDE patients included different areas of the thalamus with bilateral lateroposterior and mostly right- sided anteroventral engagement. The anteroventral/anteromedial together with the mediodorsal nuclei play important roles in connecting subcortical limbic structures (amygdala) to the limbic cortex (anterior cingulate and orbitofrontal cortex) [56]. In addition, the cerebello–thalamo–cortical loops are implicated in emotion regulation and subjective sense of control [57]; and aberrant intrinsic FC of the thalamocortical pathway was associated with depression [58].

The first negative cluster (MDE > Control), which is the same for all considered measures, includes the following brain areas: superior frontal gyrus (medial orbital), rectus gyrus, and medial and anterior orbital gyri. Thus, in the MDE group, more developed network clusters are formed near these nodes, while they are also more strongly integrated into the whole network, being large hubs (see also Figure 3). The second negative cluster is unique for the eigenvector centrality measure and includes thalamic nodes. Note that a significantly large number of positive clusters in comparing the control vs. MDE groups is a characteristic feature of the functional network.

The orbitofrontal cortex (OFC) plays a key role in emotion, by representing the reward value of the goals for action [59]. Its involvement in depression has been supported by a number of structural and functional imaging studies [10,60]. The ENIGMA consortium found that MDD patients had thinner gray matter in OFC [61]. Moreover, one of the most recent hypotheses about the development of depression, namely the non-reward attractor theory of depression, assigns a major role of the lateral OFC in the pathophysiology of the disorder [62]. According to the author the lateral orbitofrontal cortex non-reward system triggers negative cognitive states, which in turn have positive feedback top-down effects on the orbitofrontal cortex non-reward system. Our results of increased network measures of the anterior OFC (corresponding to the functional division of lateral OFC] hub in depressed patients is in line with this recent theory.

On the other hand, we know that the medial OFC is activated by rewarding and subjectively pleasant stimuli and MDD has been found to be characterized by reduced functional connectivity of the mOFC with the parahippocampal gyrus, which is in line with the clinical manifestations of anhedonia (reduced anticipatory and consummatory pleasure [63]. Our results support previous findings of depression being characterized by disturbances of both lateral and medial part of the OFC.

The most important to the clinical reality findings of the current study was derived from the linear discriminant analysis. It demonstrated that the clustering coefficient was the most ineffective measure, while full-connectivity matrices, as well as those with only the significant connections identified in advance, were the most precise in differentiating between depression in patients and healthy individuals. These measures reached precision levels of 97% and 94%, respectively. Thus, the connectivity matrices outperformed the network-specific features of node strength and node centrality. We can speculate that in order to demonstrate a meaningful diagnostic value, the resting-state connectivity feature of depression should be considered as a whole and not reduced to separate network measures.

Earlier connectivity-based classification studies focused on specific regions or networks, some of them reaching very good accuracy levels of around 90% [64]. Later, whole-brain connectivity analysis was favored, and the prediction levels reached 94% [65]. Some of the most recent studies adopted a fusion strategy using different connectivity features (intrinsic, dynamic FC, effective connectivity) that could distinguish between MDD and controls with an accuracy of 90.91% and an AUC of 0.895 [66]. In this regard, our study yielded one of the highest performances of the classifier based on whole-brain functional connectivity analysis.

## 5. Limitations

This study has several limitations that must be admitted. Firstly, the sample size is relatively small, although comparable to other single-site studies. Secondly, the patient group is heterogeneous in terms of diagnosis (major depressive and bipolar disorder) and this may have influenced the results since both common and distinct activity and connectivity patterns have been demonstrated [67,68]. However, we have studied dysfunctional connectivity as a state dependent measure in major depressive episode. Unlike the trait (or state-independent indicators), clinical features as well as the underlying functional networks impairment on the level of the episode or the syndrome are more or less shared between BAD and MDD. Thirdly, the fact that all patients have been on stable antidepressant medication prior to inclusion might have contributed as well to our findings. Future research should address those limitations by increasing the number of subjects, exploring the two types of depression separately, and including samples of non-medicated patients.

## 6. Conclusions

In the recent decade, there is a major paradigm shift toward the network-connectivity-driven classification of mental disorders [69]. Our work contributes to this field by providing insights into impairment of resting-state network hubs elucidated by functional connectivity measures such as node centrality, node strength and clustering coefficient. This approach delineated brain structures, including the lingual gyrus, cerebellum, midcingulate cortex and thalamus, as being more prominent in healthy subjects as compared to depression where the orbitofrontal cortex emerged as a key node. In addition, the connectivity matrices proved to be suitable for differentiating between patients with MDE and HC with fairly high precision levels. Further integration of these results with clinical measures, structural and diagnostic task-related functional MRI on the level of multivariate analysis may outline new approaches and criteria to the definition of mental disorders [70,71,72].

## Figures and Tables

**Table 1:** Demographic and clinical characteristics of the groups.

	Healthy Controls (*n* = 49)	Patients (*n* = 41)	Statistic	Significance
**Age** (mean ± SD)	40.7 ± 13.5	41.2 ± 15.4	D-statistic = 0.121	0.842 ^a^
**Sex** (M/F)	14/35	14/27	χ^2^-statistic = 0.324	0.569 ^b^
**Education** (secondary/higher)	16/33	20/21	χ^2^-statistic = 2.419	0.120 ^b^
**MADRS score** (mean ± SD)	2.2 ± 2.9	29.8 ± 8.2	D-statistic = 0.975	* 10^−22 a^

SD—Standard Deviation, ^a^ Two-sample Kolmogorov–Smirnov nonparametric test, ^b^ χ^2^-test, MADRS—Montgomery–Åsberg Depression Rating Scale, * *p* < 0.05.

**Table 2:** The top 30 significant connections were stronger in the control group compared to the depression group.

	Connection			Connectivity t-Statistic
1	Precuneus_L	—	Precuneus_R	31.3
2	Cuneus_L	—	Cuneus_R	28.6
3	Cerebellum_4_5_L	—	Cerebellum_4_5_R	28.3
4	Cuneus_R	—	Occipital_Sup_R	24.7
5	Cuneus_L	—	Occipital_Sup_L	24.2
6	Cerebellum_4_5_L	—	Vermis_4_5	21.0
7	Cerebellum_6_L	—	Cerebellum_6_R	20.9
8	Calcarine_R	—	Cuneus_R	20.8
9	Cuneus_R	—	Occipital_Sup_L	20.7
10	Hippocampus_L	—	Hippocampus_R	19.1
11	Calcarine_R	—	Cuneus_L	18.0
12	Cerebellum_4_5_L	—	Cerebellum_6_L	17.3
13	Calcarine_R	—	Occipital_Mid_R	16.7
14	Caudate_L	—	Caudate_R	16.6
15	Cerebellum_3_R	—	Cerebellum_4_5_R	16.5
16	Fusiform_R	—	Cerebellum_4_5_L	16.5
17	Cerebellum_4_5_R	—	Vermis_4_5	16.3
18	Fusiform_R	—	Vermis_4_5	16.3
19	Cingulate_Mid_L	—	Cingulate_Mid_R	16.0
20	Occipital_Sup_R	—	Occipital_Mid_L	15.8
21	Hippocampus_R	—	Temporal_Mid_R	15.5
22	Cuneus_R	—	Occipital_Mid_L	15.2
23	Lingual_L	—	Lingual_R	15.2
24	Hippocampus_L	—	Cerebellum_4_5_L	14.5
25	Fusiform_R	—	Cerebellum_4_5_R	14.4
26	Cerebellum_4_5_R	—	Cerebellum_6_L	14.3
27	Thal_MDm_L	—	Thal_MDl_L	14.2
28	Cerebellum_4_5_R	—	Cerebellum_6_R	13.8
29	Fusiform_R	—	Cerebellum_6_L	13.8
30	Calcarine_L	—	Calcarine_R	13.6

**Table 3:** Significant clusters for the node eigenvector centrality measure; here, superscripts “+” and “−” denote positive (Control > MDE) and negative (MDE > Control) clusters, respectively.

Cluster	Nodes	Region	*p*
1^+^	49	Lingual L	0.001
	50	Lingual R	
	51	Occipital Sup L	
	52	Occipital Sup R	
	53	Occipital Mid L	
	54	Occipital Mid R	
2^+^	109	Vermis 1, 2	0.005
	110	Vermis 3	
	111	Vermis 4, 5	
	112	Vermis 6	
	113	Vermis 7	
3^+^	35	Cingulate Mid L	0.009
	36	Cingulate Mid R	
	37	Cingulate Post L	
4^+^	118	Thal AV R	0.014
	119	Thal LP L	
	120	Thal LP R	
5^+^	7	Frontal Inf Oper L	0.022
	8	Frontal Inf Oper R	
	9	Frontal Inf Tri L	
1^−^	21	Frontal Med Orb L	0.001
	22	Frontal Med Orb R	
	23	Rectus L	
	24	Rectus R	
	25	OFCmed L	
	26	OFCmed R	
	27	OFCant L	
	28	OFCant R	
2^−^	121	Thal VA L	0.006
	122	Thal VA R	
	123	Thal VL L	
	124	Thal VL R	

**Table 4:** Significant clusters for the node strength measure; here, superscripts “+” and “−” denote positive (Control > MDE) and negative (MDE > Control) clusters, respectively.

Cluster	Nodes	Region	*p*
1^+^	47	Cuneus L	0.005
	48	Cuneus R	
	49	Lingual L	
	50	Lingual R	
	51	Occipital Sup L	
	52	Occipital Sup R	
	53	Occipital Mid L	
2^+^	109	Vermis 1, 2	0.029
	110	Vermis 3	
	111	Vermis 4, 5	
	112	Vermis 6	
	113	Vermis 7	
3^+^	35	Cingulate Mid L	0.050
	36	Cingulate Mid R	
	37	Cingulate Post L	
4^+^	118	Thal AV R	0.050
	119	Thal LP L	
	120	Thal LP R	
1^−^	21	Frontal Med Orb L	0.003
	22	Frontal Med Orb R	
	23	Rectus L	
	24	Rectus R	
	25	OFCmed L	
	26	OFCmed R	
	27	OFCant L	
	28	OFCant R	

**Table 5:** Significant clusters for the clustering coefficient measure; here, superscripts “+” and “−” denote positive (Control > MDE) and negative (MDE > Control) clusters, respectively.

Cluster	Nodes	Region	*p*
1^+^	47	Cuneus L	0.009
	48	Cuneus R	
	49	Lingual L	
	50	Lingual R	
	51	Occipital Sup L	
	52	Occipital Sup R	
	53	Occipital Mid L	
2^+^	109	Vermis 1, 2	0.035
	110	Vermis 3	
	111	Vermis 4, 5	
	112	Vermis 6	
	113	Vermis 7	
1^−^	21	Frontal Med Orb L	0.003
	22	Frontal Med Orb R	
	23	Rectus L	
	24	Rectus R	
	25	OFCmed L	
	26	OFCmed R	
	27	OFCant L	
	28	OFCant R	

**Table 6:** Classification accuracy of different connectivity features.

#	Feature Vector	Accuracy (Mean ± SD)	Sensitivity	Specificity	Precision
1	Full FC matrices	0.9278 ± 0.0102	84%	99%	98%
2	FC matrices with significant connections	0.8954 ± 0.0232	83%	92%	90%
3	Clustering coefficient	0.8425 ± 0.0263	87%	81%	79%
4	Node strength	0.8382 ± 0.0181	87%	83%	80%
5	Eigenvector centrality	0.8519 ± 0.0197	81%	89%	86%

Gray line highlights the unsuitable feature for classification.

## Data Availability

All data is available from the authors upon reasonable request.

## Appendix A

**Table A1:** List of significant (found with the FDR method) connections between positive clusters nodes (See Figure 2) for the node centrality measure.

Region 1		Region 2
‘Frontal Inf Oper L’	—	‘Frontal Inf Oper R’
‘Frontal Inf Oper L’	—	‘Frontal Inf Tri L’
‘Frontal Inf Oper L’	—	‘Cingulate Mid L’
‘Frontal Inf Oper L’	—	‘Cingulate Mid R’
‘Frontal Inf Oper L’	—	‘Cuneus L’
‘Frontal Inf Oper L’	—	‘Cuneus R’
‘Frontal Inf Oper L’	—	‘Lingual L’
‘Frontal Inf Oper L’	—	‘Lingual R’
‘Frontal Inf Oper L’	—	‘Occipital Sup L’
‘Frontal Inf Oper L’	—	‘Occipital Sup R’
‘Frontal Inf Oper L’	—	‘Occipital Mid R’
‘Frontal Inf Oper L’	—	‘Vermis 3’
‘Frontal Inf Oper L’	—	‘Vermis 4, 5’
‘Frontal Inf Oper L’	—	‘Vermis 7’
‘Frontal Inf Oper L’	—	‘Thal LP L’
‘Frontal Inf Oper R’	—	‘Frontal Inf Tri L’
‘Frontal Inf Tri L’	—	‘Cingulate Mid L’
‘Frontal Inf Tri L’	—	‘Cingulate Mid R’
‘Frontal Inf Tri L’	—	‘Cingulate Post L’
‘Frontal Inf Tri L’	—	‘Cuneus L’
‘Frontal Inf Tri L’	—	‘Cuneus R’
‘Frontal Inf Tri L’	—	‘Lingual R’
‘Frontal Inf Tri L’	—	‘Occipital Sup L’
‘Frontal Inf Tri L’	—	‘Occipital Sup R’
‘Frontal Inf Tri L’	—	‘Occipital Mid L’
‘Frontal Inf Tri L’	—	‘Occipital Mid R’
‘Frontal Inf Tri L’	—	‘Vermis 4, 5’
‘Frontal Inf Tri L’	—	‘Vermis 6’
‘Frontal Inf Tri L’	—	‘Vermis 7’
‘Cingulate Mid L’	—	‘Cingulate Mid R’
‘Cingulate Mid L’	—	‘Cingulate Post L’
‘Cingulate Mid L’	—	‘Vermis 3’
‘Cingulate Mid L’	—	‘Vermis 7’
‘Cingulate Mid R’	—	‘Cingulate Post L’
‘Cingulate Post L’	—	‘Cuneus L’
‘Cingulate Post L’	—	‘Cuneus R’
‘Cingulate Post L’	—	‘Lingual L’
‘Cingulate Post L’	—	‘Lingual R’
‘Cingulate Post L’	—	‘Occipital Sup L’
‘Cingulate Post L’	—	‘Occipital Sup R’
‘Cingulate Post L’	—	‘Occipital Mid L’
‘Cingulate Post L’	—	‘Occipital Mid R’
‘Cingulate Post L’	—	‘Vermis 3’
‘Cingulate Post L’	—	‘Vermis 4, 5’
‘Cingulate Post L’	—	‘Vermis 6’
‘Cingulate Post L’	—	‘Vermis 7’
‘Cuneus L’	—	‘Cuneus R’
‘Cuneus L’	—	‘Lingual L’
‘Cuneus L’	—	‘Lingual R’
‘Cuneus L’	—	‘Occipital Sup L’
‘Cuneus L’	—	‘Occipital Sup R’
‘Cuneus L’	—	‘Occipital Mid L’
‘Cuneus L’	—	‘Occipital Mid R’
‘Cuneus L’	—	‘Vermis 3’
‘Cuneus L’	—	‘Vermis 4, 5’
‘Cuneus L’	—	‘Vermis 6’
‘Cuneus L’	—	‘Vermis 7’
‘Cuneus R’	—	‘Lingual L’
‘Cuneus R’	—	‘Lingual R’
‘Cuneus R’	—	‘Occipital Sup L’
‘Cuneus R’	—	‘Occipital Sup R’
‘Cuneus R’	—	‘Occipital Mid L’
‘Cuneus R’	—	‘Occipital Mid R’
‘Cuneus R’	—	‘Vermis 3’
‘Cuneus R’	—	‘Vermis 4, 5’
‘Cuneus R’	—	‘Vermis 6’
‘Cuneus R’	—	‘Vermis 7’
‘Lingual L’	—	‘Lingual R’
‘Lingual L’	—	‘Occipital Sup L’
‘Lingual L’	—	‘Occipital Sup R’
‘Lingual L’	—	‘Occipital Mid L’
‘Lingual L’	—	‘Occipital Mid R’
‘Lingual L’	—	‘Vermis 1, 2’
‘Lingual L’	—	‘Vermis 3’
‘Lingual L’	—	‘Vermis 4, 5’
‘Lingual L’	—	‘Vermis 7’
‘Lingual R’	—	‘Occipital Sup L’
‘Lingual R’	—	‘Occipital Sup R’
‘Lingual R’	—	‘Occipital Mid L’
‘Lingual R’	—	‘Occipital Mid R’
‘Lingual R’	—	‘Vermis 1, 2’
‘Lingual R’	—	‘Vermis 3’
‘Lingual R’	—	‘Vermis 4, 5’
‘Lingual R’	—	‘Vermis 6’
‘Lingual R’	—	‘Vermis 7’
‘Occipital Sup L’	—	‘Occipital Sup R’
‘Occipital Sup L’	—	‘Occipital Mid L’
‘Occipital Sup L’	—	‘Occipital Mid R’
‘Occipital Sup L’	—	‘Vermis 3’
‘Occipital Sup L’	—	‘Vermis 4, 5’
‘Occipital Sup L’	—	‘Vermis 6’
‘Occipital Sup L’	—	‘Vermis 7’
‘Occipital Sup R’	—	‘Occipital Mid L’
‘Occipital Sup R’	—	‘Occipital Mid R’
‘Occipital Sup R’	—	‘Vermis 3’
‘Occipital Sup R’	—	‘Vermis 4, 5’
‘Occipital Sup R’	—	‘Vermis 6’
‘Occipital Sup R’	—	‘Vermis 7’
‘Occipital Mid L’	—	‘Occipital Mid R’
‘Occipital Mid L’	—	‘Vermis 3’
‘Occipital Mid L’	—	‘Vermis 4, 5’
‘Occipital Mid L’	—	‘Vermis 6’
‘Occipital Mid L’	—	‘Vermis 7’
‘Occipital Mid R’	—	‘Vermis 3’
‘Occipital Mid R’	—	‘Vermis 4, 5’
‘Occipital Mid R’	—	‘Vermis 6’
‘Occipital Mid R’	—	‘Vermis 7’
‘Vermis 1 2’	—	‘Vermis 3’
‘Vermis 3’	—	‘Vermis 4, 5’
‘Vermis 3’	—	‘Vermis 6’
‘Vermis 3’	—	‘Vermis 7’
‘Vermis 4 5’	—	‘Vermis 6’
‘Vermis 4 5’	—	‘Vermis 7’
‘Vermis 6’	—	‘Vermis 7’

**Table A2:** List of significant (found with the FDR method) between negative clusters nodes (See Figure 3) for the node centrality measure.

Region 1		Region 2
‘Frontal Med Orb L’	—	‘Frontal Med Orb R’
‘Frontal Med Orb L’	—	‘Rectus L’
‘Frontal Med Orb L’	—	‘Rectus R’
‘Frontal Med Orb L’	—	‘OFCmed L’
‘Frontal Med Orb L’	—	‘OFCmed R’
‘Frontal Med Orb L’	—	‘OFCant L’
‘Frontal Med Orb L’	—	‘OFCant R’
‘Frontal Med Orb L’	—	‘Thal VA L’
‘Frontal Med Orb L’	—	‘Thal VA R’
‘Frontal Med Orb R’	—	‘Rectus L’
‘Frontal Med Orb R’	—	‘Rectus R’
‘Frontal Med Orb R’	—	‘OFCmed L’
‘Frontal Med Orb R’	—	‘OFCmed R’
‘Frontal Med Orb R’	—	‘OFCant L’
‘Frontal Med Orb R’	—	‘OFCant R’
‘Frontal Med Orb R’	—	‘Thal VA L’
‘Frontal Med Orb R’	—	‘Thal VA R’
‘Rectus L’	—	‘Rectus R’
‘Rectus L’	—	‘OFCmed L’
‘Rectus L’	—	‘OFCmed R’
‘Rectus L’	—	‘OFCant L’
‘Rectus L’	—	‘OFCant R’
‘Rectus L’	—	‘Thal VA L’
‘Rectus L’	—	‘Thal VA R’
‘Rectus L’	—	‘Thal VL L’
‘Rectus L’	—	‘Thal VL R’
‘Rectus R’	—	‘OFCmed L’
‘Rectus R’	—	‘OFCmed R’
‘Rectus R’	—	‘OFCant L’
‘Rectus R’	—	‘OFCant R’
‘Rectus R’	—	‘Thal VA L’
‘Rectus R’	—	‘Thal VA R’
‘Rectus R’	—	‘Thal VL L’
‘Rectus R’	—	‘Thal VL R’
‘OFCmed L’	—	‘OFCmed R’
‘OFCmed L’	—	‘OFCant L’
‘OFCmed L’	—	‘OFCant R’
‘OFCmed L’	—	‘Thal VA L’
‘OFCmed L’	—	‘Thal VA R’
‘OFCmed L’	—	‘Thal VL L’
‘OFCmed L’	—	‘Thal VL R’
‘OFCmed R’	—	‘OFCant L’
‘OFCmed R’	—	‘OFCant R’
‘OFCmed R’	—	‘Thal VA L’
‘OFCmed R’	—	‘Thal VA R’
‘OFCmed R’	—	‘Thal VL L’
‘OFCmed R’	—	‘Thal VL R’
‘OFCant L’	—	‘OFCant R’
‘OFCant L’	—	‘Thal VA L’
‘OFCant L’	—	‘Thal VA R’
‘OFCant L’	—	‘Thal VL R’
‘OFCant R’	—	‘Thal VA L’
‘OFCant R’	—	‘Thal VA R’
‘OFCant R’	—	‘Thal VL L’
‘Thal VA R’	—	‘Thal VL L’
